# Iodine-125 Seeds Inhibit Carcinogenesis of Hepatocellular Carcinoma Cells by Suppressing Epithelial-Mesenchymal Transition via TGF-*β*1/Smad Signaling

**DOI:** 10.1155/2022/9230647

**Published:** 2022-05-07

**Authors:** Chongshuang Yang, Yunhua Xiao, Yexiang Du, Junru Xiong, Liangyu Deng, Qinghua Liang, Jing Yuan, Chuang He, Fengtian He, Xuequan Huang

**Affiliations:** ^1^Treatment Center of Minimally Invasive Intervention and Radioactive Particles, First Affiliated Hospital of the Army Medical University, Chongqing, China; ^2^Department of Digestive Medicine, The Second Affiliated Hospital of Army Medical University, Army Medical University, Chongqing, China; ^3^Department of Radiology, The Third Affiliated Hospital of Army Medical University, Army Medical University, Chongqing, China; ^4^Department of Biochemistry and Molecular Biology, College of Basic Medical Sciences, Army Medical University, Chongqing, China

## Abstract

To investigate the radioactive iodine-125 (I-125) seed on migrating and invading of hepatocellular carcinoma (HCC) cells and its mechanism, the irradiation of PLC and Huh7 cells was carried out with I-125 seeds in vitro. Cell counting kit 8 assay was employed to measure cell viability. Cell migration was evaluated by using wound-healing assay. Cell invasion was detected by Transwell assay; RT-PCR and Western blot were used for the detection of the mRNA and proteins of TGF-*β*1 signaling pathway-related genes. The viability of PLC and Huh7 cells declined in a dose-dependent manner with increasing irradiation from 0 Gy, 2 Gy, 4 Gy, and 6 Gy, to 8 Gy, respectively. The IC50 of PLC and Huh7 cells were 6.20 Gy and 5.39 Gy, respectively, after 24 h of irradiation. Migration and invasion abilities of I-125 group cells were greatly weakened (*P* < 0.05) comparing with the control group. According to the outcomes of RT-PCR and WB, I-125 seed irradiation significantly inhibited the mRNA and protein expression of N-cadherin, vimentin, TGF-*β*1, p-Smad2/3, and Snail. But the mRNA and protein expressions of E-cadherin were enhanced. Rescue experiment demonstrates that TGF-*β*1 activator could reverse the inhibitory effects of I-125 on invasion and migration of cells. The results of in vivo experiments further verified that the I-125 seeds can inhibit the proliferation and TGF-*β*1 of xenographed PLC cells. In conclusion, I-125 seeds restrain the invasion and migration of HCC cells by suppressing epithelial to mesenchymal transition, which may associate with the inhibition of the TGF-*β*1 signaling.

## 1. Introduction

As one of the most common malignant tumors across the world, hepatocellular carcinoma (HCC) is at the forefront in both incidence rate and mortality rate [1, 2]. Surgical resection is still the first option for the stage I or II of HCC. However, typical clinical symptoms can hardly be found in the patient in the early stage of HCC, and most of them were in the middle and late stages when diagnosed. About 70% patients lost the chance to have surgery when they are diagnosed with HCC [1]. Sorafenib, a multienzyme inhibitor, are somehow effective to HCC. But what needs to be mentioned is its drug resistance [3]. At present, there is no effective treatment for advanced HCC [4]. Metastasis of cancer cells mainly explains the failure of treatment, and it is also one of the biggest challenges in the treatment of HCC.

Epithelial-mesenchymal transition (EMT) means the process that epithelial-like cells transform into mesenchymal-like cells under the stimulation of various factors, which has been considered as essential for tumor cell metastasis [5]. The epithelial cells lost polarity after EMT, which leads the links between cells to be interrupted; the movement of cells are enhanced. Finally, the tumor cells invade surrounding tissues and even metastases to other organs [6]. There are various signaling pathways regulate EMT, such as the Wnt/*β*-catenin pathway, TGF-*β*1 pathway, and NF-*κ*B Pathway [5]. The TGF-*β*1 pathway is one of the classical signaling pathways, functioning vital in the occurrence and development of tumor [7]. A large number of studies showed that the TGF-*β*1 signaling pathway regulates EMT of a variety of tumors and promotes the metastasis of tumor cells [7, 8].

With endogenous radiotherapy, I-125 radioactive seed implantation possesses exact curative effects, including high precision, strong conformability, and repeatability, which lead to fewer complications [9, 10]. The I-125 seed is one of the most used particle sources, extensively applied in treating different solid tumors. Previous studies have shown that I-125 seed implantation therapy can greatly enhance the survival rate of patients suffering from advanced HCC, especially in those with portal vein tumor thrombus [11, 12]. Also, the therapy can be used for other cancer patients who had failed or recurred [13].

Nevertheless, the mechanism of I-125 seeds in treating HCC is not fully elucidated, and especially, the effects of I-125 seeds on the EMT and TGF-*β*1 signaling pathway in HCC cells have not been investigated. In this study, we explored the function of correct dose of I-125 seed, which may generates certain degree of reactive oxygen species (ROS) inside cells, and in turn to trigger the process of anti-invasion/antimetastasis of HCC by downregulating EMT through the TGF-*β*1/Smad signaling pathway. We may provide an experimental basis for promoting the I-125 seed implantation therapy and using it as a more effective therapy for HCC.

## 2. Materials and Methods

### 2.1. Materials

#### 2.1.1. Cell Lines

PLC/PRF/5 and Huh7 cells were purchased from American Type Culture Collection (ATCC); these human HCC cell lines were incubated in modified Eagle's medium (MEM) (Hyclone, USA) by using 10% fetal bovine serum (FBS) (Hyclone, USA) at the temperature of 37°C and 5% CO_2_. Both PLC and Huh7 cell lines are sensitive to reactive oxygen species (ROS) because they are widely used in an antioxidant assay.

#### 2.1.2. Irradiation Model of I-125 Seeds

**Figure 1:**
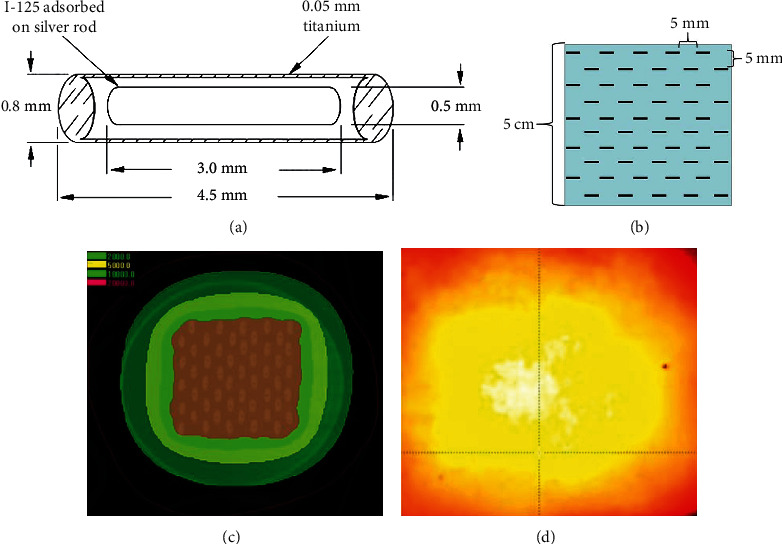
Iodine-125 seed irradiation model in vitro. (a) The I-125 seed structural representation. (b) The arrangement of 125I seeds on the surface of seed plate. (c) The irradiation model was verified by treatment planning system. (d) The irradiation model was verified by the film calibration radiation dose method.

I-125 seeds (Beijing Zhibo Hi-tech Biotechnology Co., Ltd., China) as shown in schematic digram (Figure 1(a)) were set on the surface of the plate within the appliance, and the seed plate with seeds was set on the support plate above the cell dish. Distance from the bottom of cell dish to the bottom of the seed plate was 5 mm. Those devices were placed in a square container made of 3 mm thick lead, and the wall of the container has four ventilation holes. Subsequently, when in use, 50 I-125 seeds are placed in a square with a side length of 50 mm evenly, and the transverse and longitudinal distance between seeds is 5 mm **(**Figure 1(b)**)**. The initial activity of iodine 125 seeds was 0.8 mCi. The initial average dose and the dose distribution of each point on the cell dish were calculated by using Monte Carlo model. The treatment planning system (TPS) and the film calibration radiation dose method are used to evaluate the uniformity of the radiation dose distribution at different points on the bottom of the cell dish **(**Figures 1(c) and 1(d)**)**. The total radiation dose within a specific exposure time or the time (*t*) required to reach a specific radiation dose can be calculated by the following formula [14]:

(1) $$\begin{array}{c} D_{t}=D_{0}\int_{t=0}^{t=T}e^{-\lambda t}dt, \end{array}$$

where *D*_0_ is the initial dose rate. *T* is a half-life, equal to 1425.6 hours (59.6 days). *λ* is the decay constant of iodine 125 seeds, and *t* is the irradiation time (hours).

## 3. Experimental Methods

### 3.1. I-125 Seed Irradiation on PLC and Huh7 Cells

I-125 seed irradiation at a dose of 0, 2, 4, 6, and 8*Gy* was used to treat PLC and Huh7 cells, respectively. During the irradiation, the module was always placed in the 37°C cell incubator. No treatment for the control group, the I-125 group was treated with 4 Gy irradiation, and I-125+TGF-*β*1 group was treated with 4 Gy irradiation and TGF-*β*1 activator (10 *μ*g/ml) (MedChem Express, Cat. SRI-011381, Monmouth, NJ, USA).

### 3.2. Cell Viability Assay

PLC and Huh7 cell lines of HCC have been shown to be well responded to reactive oxygen species (ROS), and I-125 irradiation universally generates ROS in the cellular mitochondria. Cell viabilit**y** of PLC and Huh7 cells was conducted using a CCK8 kit (Beyotime, China). Briefly, PLC and Huh7 cells were collected after treating with varied doses (0, 2, 4, 6, and 8 Gy) irradiation. Subsequently, cell density was adjusted to 4 × 10^4^ cells/ml; then, 100 *μ*l/well cell suspension was added into a 96 well plate, respectively, and incubated for 24 h at 37°C with 5% CO_2_. Three multiple holes were set in each group. Each well was added with CCK8 (10 *μ*l) solution, and the measurement of absorbance at 450 nm was made.

### 3.3. Wound Healing Assay

PLC and Huh7 cells were pretreated with 0 Gy and 4 Gy dose irradiation, respectively, at 80% confluence and scraped through the monolayer cells with a 10 *μ*l micropipette tip to make wounds. After being washed with PBS, the cancer cells were cultured in serum-free medium. A microscope was used to visually observe and measure the wound distance (Olympus, Japan), at immediately and 24 h later.

### 3.4. Migration and Invasion Assay

Different doses (0.4Gy) of irradiation were used to treat PLC and Huh7 cells; migration and invasion assay was conducted following the commercial protocol. Briefly, cell density was adjusted to 1 × 10^6^ cells/ml. Later, the upper chamber of Transwell (Corning, USA) was added with 200 *μ*l of cell suspension, and the 24-hour incubation of the lower chamber added with 500 *μ*l of medium with 10% FBS was made at 37°Cwith 5% CO_2_. The culture medium was discarded after being washed with PBS; cells were fixed with 4% of paraformaldehyde and stained with 0.1% of crystal violet. The random selection of five fields for photographing and counting was made. The difference between migration assay and invasion assay was whether the bottom of Transwell is pretreated with diluted Matrigel matrix, or without (Corning, New York, USA).

### 3.5. Western Blotting Assay

The extraction of total protein was made with RIPA lysate buffer and protease inhibitor, and its concentration was determined by bicinchoninic acid (BCA) assay kit (Beyotime, China). 10% SDS-PAGE (Beyotime, China) separated protein which then transferred to the PVDF membrane. Later, 5% skimmed milk at room temperature was used to block the membrane for 1 h. Following, primary antibodies against E-cadherin, N-cadherin, vimentin (CST, Danvers, MA, USA), TGF-*β*1, Smad2, and Smad3 (Proteintech Group, USA), p-Smad2, and p-Smad3 (Affinity, Affbiotech Group, USA) were added and incubated overnight at 4°C. Horseradish peroxidase-labeled second antibody (CST, Danvers, MA, USA) was incubated for 1 hour. Protein was detected by enhanced chemiluminescence.

### 3.6. RT-PCR

After extracted by TRIzol (Takara, Japan), total RNA was transcribed into cDNA reversely. Then, the QRT-PCR was made with SYBR Green fluorescent dye method using cDNA as template. The reaction system was SYBR Green 10 *μ*l; all primers were ordered from Sangon Biotech (Shanghai, China) 0.8 *μ*l, ddH_2_O 7.2 *μ*l, and cDNA 2 *μ*l; and the total system was 20 *μ*l. The qPCR procedure was as follows: 95°C 30 s; 95°C 5 s, 60°C 30 s, 72°C 15 s, 40 cycles; 95°C 15 s, 60°C 1 min, and 95°C 15 s. The expression difference of each gene was compared by 2^-∆∆CT^ relative quantitative method (∆CT : the CT value of the target gene − CT value of GAPDH Gene, ∆∆CT value = the∆CT value of the experiment group − the∆CT value of the control group). The expression of gene in control group is 1, and the relative expression of gene in the experimental group is 2^-∆∆CT^. The primer sequences are shown in Table 1.

### 3.7. Immunofluorescence Assay

In short, the required cells were seeded on the glass slide. The cells seeded on the glass slide were washed after 24 h of incubation, fixed, and infiltrated for 10 minutes and then blocked with 5% of bovine serum albumin (BSA) for 30 minutes. Subsequently, the antitarget protein antibody E-cadherin, N-cadherin, TGF-*β*1, and p-Smad3 solution was added and incubated at 4°C overnight. Alexa Fluor 549-conguated secondary antibody (Invitrogen, USA) was used. 4′, 6′-diamino-2-phenylindole (DAPI) (Invitrogen, USA) was adopted to stain cell nuclei. The images were recorded and photographed with a fluorescence microscope (Olympus, Japan).

### 3.8. Nude Mouse Model and Immunohistochemistry Assay

Ten nude mice (4-6 weeks old) were purchased from local university facility. The subcutaneous inoculation of PLC cells (1 × 10^7^ cells/ml) was made to these nude mice. About 10 days later, the subcutaneous transplanted tumor in nude mice was about 250-300 mm^3^. The nude mice were randomly fallen into the control group and I-125 group, with 5 nude mice in each group. In the I-125 group, a 0.8 mCi I-125 seed was implanted into each subcutaneously transplanted tumor center. But the control group was untreated. Then, the length (*a*) and width (*b*) of the tumor was measured every four days, and the calculation of tumor volume (*V*) as the following formula: *V* = 1/2 *a*.*b*^2^ was made. After 28 days, mice were euthanized, and the collection of tumors was conducted for further study. The ethics committee of the First Affiliated Hospital of Army Medical University approved all experimental protocols.

After being fixed with 4% paraformaldehyde, xenograft tumor tissues were embedded in paraffin and sectioned. Then, the antibodies against TGF-*β*1 were stained with DAB horseradish peroxidase chromogenic Kit (Beyotime, China) at room temperature. After being stained with hematoxylin for 30 s, sections were dehydrated and fixed and then sealed with neutral glue. And a microscope was used to observe and photograph all stained images (Olympus, Japan).

### 3.9. Statistical Analysis

The experimental data and image preprocessing were analyzed by SPSS24.0 (IBM, USA) and GraphPad prism 6.0 software. The significant distinction between the two groups was analyzed by employing the independent *t*-test, and the distinctions among the groups were analyzed by adopting one-way ANOVA. *P* < 0.05 or *P* < 0.01 was of statistical significance.

## 4. Results

### 4.1. I-125 Seed Irradiation Suppresses HCC Proliferation, Migration, and Invasion

**Figure 2:**
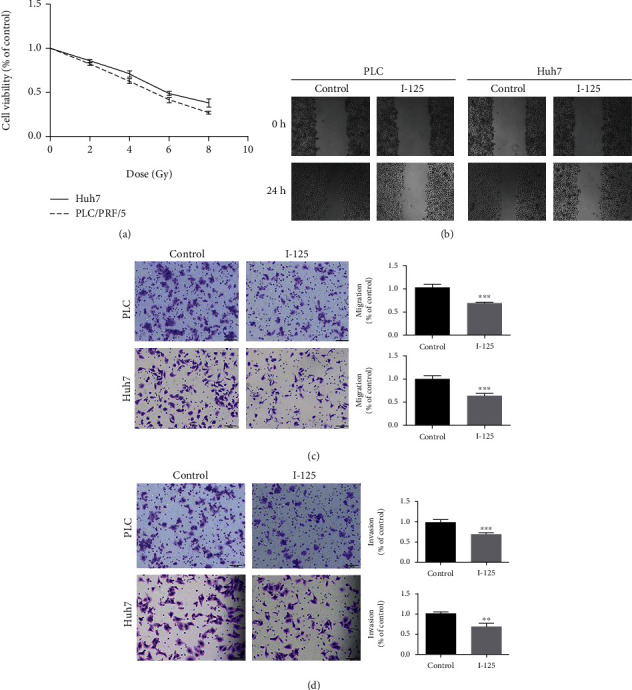
I-125 seed irradiation suppresses cell proliferation, migration, and invasion of PLC and Huh7. (a) Cell viability of PLC and Huh7 cells was evaluated after 24 h of incubation with I-125 seed irradiation at varied doses (0, 2, 4, 6, and 8 Gy). Migration capabilities of PLC and Huh7 cells with the treatment of 0 and 4 Gy I-125 irradiation was assessed with wound healing assay (b) and migration assay (c). Invasive capabilities of PLC and Huh7 cells with the treatment of 0 and 4 Gy I-125 irradiation was assessed with Transwell assay (d). The average number of invasive cells was calculated by counting the number of cells in 5 fields per chamber. The expression of data as the mean ± standard error (*n* = 3); ^∗^*P* < 0.05 vs. the control group.

The dose of I-125 for irradiation was chosen based on growth curve of PLC and Huh7 in normal control and I-125 incubation based on their IC50 in order to see the direct effect of I-125 on HCC proliferation, which shows the cell proliferation decreased as increased radiation dose (Figure 2(a)); the IC50 of PLC and Huh7 cells was 6.20 Gy and 5.39 Gy at 24 hours after irradiation, respectively. In order to observe the effects of I-125 seed on migration and invasion of PLC and Huh7 cells, 4 Gy was used for subsequent experiments. By visual measurement of the confluence cell edges between the strip, and comparing with the control group, the migration ability of PLC and Huh7 cells was weakened after I-125 seed irradiation, and the relative migration ability was reduced to 53% and 65%, respectively (Figures 2(b) and 2(c)**)**. In addition, the result of transwell assay indicated that the I-125 seed irradiation dramatically inhibited the invasion of PLC and Huh7 cells (*P* < 0.01, Figure 2(d)**).** All above suggest that I-125 seed irradiation inhibits cell proliferation, migration, and invasion of HCC.

### 4.2. I-125 Seed Irradiation Inhibited EMT of PLC and Huh7 Cells

**Figure 3:**
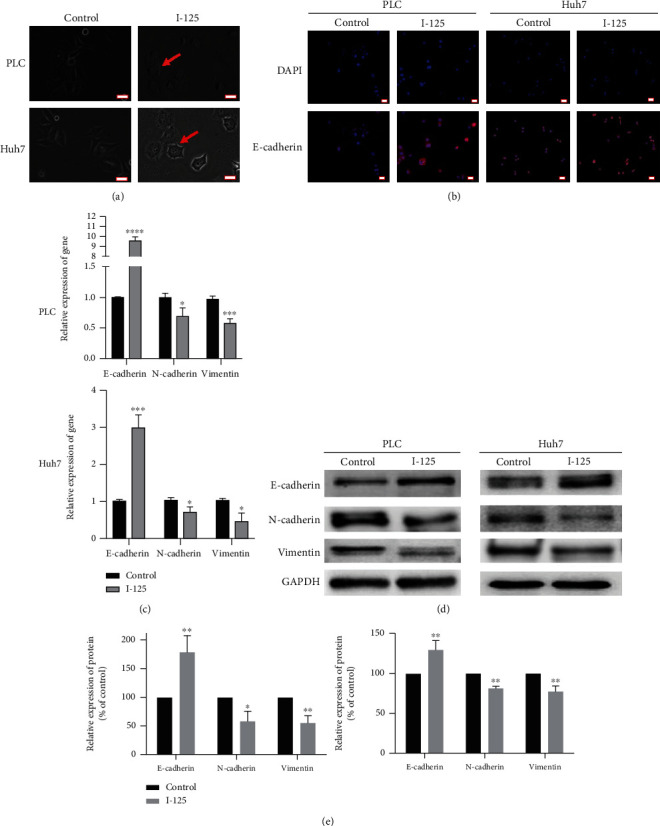
I-125 seed irradiation inhibits EMT of PLC and Huh7 cells. (a) Morphological changes of PLC (upper panel) and Huh7 (lower panel) cells after irradiation (red arrow indicates the EMT site). (b) By immunofluorescence staining, I-125 seed irradiation upregulated the expression of E-cadherin in PLC (left) and Huh7 (right) cells. (c) RT-PCR assay demonstrates that I-125 seed radiation upregulated the expression of E-cadherin but downregulate that of N-cadherin and vimentin. (d, e) WB assay indicated that I-125 seed radiation increase the expression of E-cadherin and weak that of N-cadherin and vimentin. The expression of data as the mean ± standard error of the mean (*n* = 3). ^∗^*P* < 0.05, ^∗∗^*P* < 0.01 vs. the control group. Bar = 20 *μ*m.

The morphology of polyhedral fibroblasts was replaced by typical cobblestone like epithelioid cells after I-125 seed irradiation (red arrow, Figure 3(a)), and I-125 seed irradiation also upregulated the protein expression of E-cadherin in PLC and Huh7 cells (Figure 3(b)). Moreover, the mRNA expression of E-cadherin in PLC and Huh7 cells were relatively increased, respectively; however, the mRNA relative expression of N-cadherin and vimentin were decreased (Figure 3(c)). Interestingly, the results of Western blotting assay were similar to the outcomes of RT-PCR (Figures 3(d) and 3(e)). So, these findings suggest that I-125 seed irradiation can inhibit or reverse the occurrence of EMT in PLC and Huh7 cells.

### 4.3. I-125 Seed Irradiation Inhibits TGF-*β*1 Signaling Pathway

**Figure 4:**
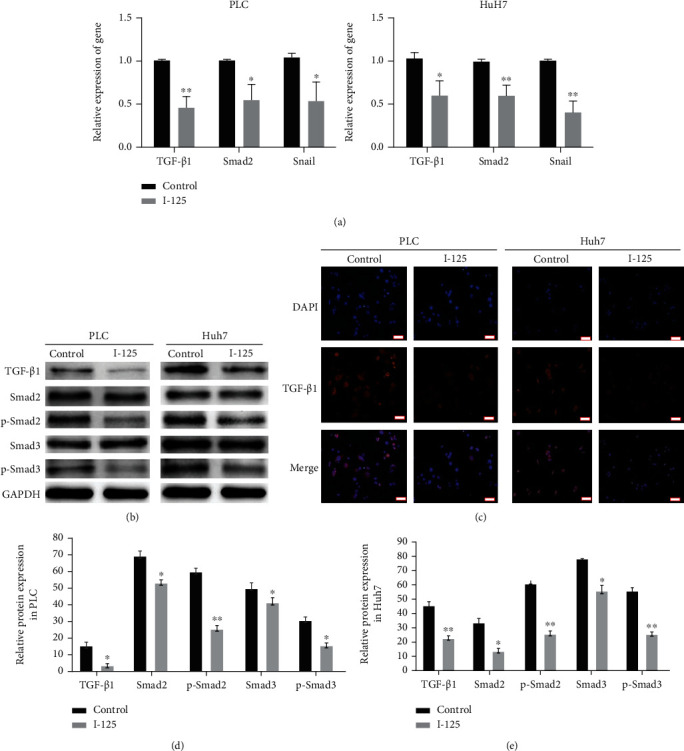
I-125 seed irradiation inhibits the TGF-*β*1 signaling pathway. (a) RT-PCR assay demonstrated that the I-125 seed radiation downregulate the expression of TGF-*β*1, Smad2, and Snail. (b). WB assay demonstrated that the I-125 seed radiation downregulate the expression of TGF-*β*1, p-Smad2, and p-Smad3. (c) By immunofluorescence staining, the I-125 seed irradiation downregulates the expression of TGF-*β*1 in PLC and Huh7 cells, respectively (data of the immunofluorescence for Smad2, p-Smad2, Smad3, and p-Smad3 not shown, bar: 20 *μ*m). (d) Relative protein expression of the genes in PLC. (e) Relative protein expression of the genes in Huh7. Data presented as the mean ± standard error of the mean (*n* = 3). ^∗^*P* < 0.05 vs. the control group.

The gene expression of TGF-*β*1, Smad2, and Snail was analyzed according to RT-PCR assays (Figure 4(a)), and the protein expression was detected by WB (Figure 4(b)) and immunofluorescent assay (Figure 4(c)); the levels of TGF-*β*1 were reduced in PLC and Huh7 cells after I-125 seed irradiation. As I-125 seed irradiation decreased the expression of TGF-*β*1, p-Smad2, and p-Smad3 (Figures 4(b), 4(d), and 4(e)) even though did not significantly affect the protein expression of Smad2 and Smad3. So, these results suggest that I-125 seed irradiation suppresses the TGF-*β*1 signaling pathway.

### 4.4. TGF-*β*1 Activator Reverses I-125 Seed Irradiation Inhibitory Effect on EMT and TGF-*β*1 Signaling Pathway

**Figure 5:**
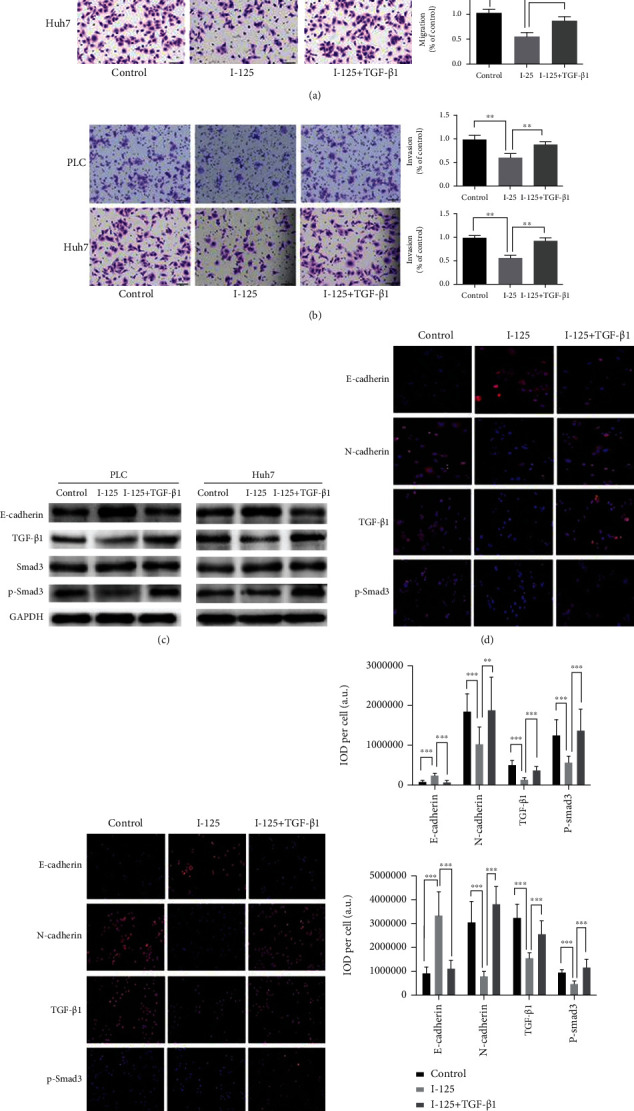
TGF-*β*1 activator reverse I-125 seed irradiation inhibitory effects on the EMT and TGF-*β*1 signaling pathway. TGF-*β*1 activator reverses the inhibitory role in migration (a) and invasion (b) of PLC and Huh7. TGF-*β*1 activator reverses the roles in the EMT and TGF-*β*1 signaling pathway-related markers by WB assay (c) and immunofluorescence assay for PLC (d) and for Huh7 (e) and their relative IOD (integrated optical density) per cell were plotted, respectively (f). The average number of invasive cells was calculated by counting then number of cells in 5 fields per chamber. Data was expressed as mean ± SD (*n* = 3). The difference was of statistical significance (*P* < 0.05).

According to the previous reports, TGF-*β* activator can activate the TGF-*β*1 signaling pathway and promote EMT; thus, obviously, TGF-*β*1 promotes tumor invasion and migration [15]. To verify the association of the inhibition of EMT of PLC and Huh7 with I-125 seed irradiated restraint of the TGF-*β*1 signaling pathway, we treated PLC and Huh7 cells with TGF-*β*1 activator (final concentration at 10 *μ*g/ml) and I-125 seed irradiation for 48 h. By comparing with the control group, the number of migration (Figure 5(a)) and invasion (Figure 5(b)) of the PLC and Huh7 cells in the I-125 group was greatly reduced (*p* < 0.05). However, in the I-125+TGF-*β*1 group, TGF-*β*1 activator partially reversed the I-125 seed irradiation inhibition effects on PLC and Huh7. Meanwhile, the protein expressions of E-cadherin, TGF-*β*1, Smad3, and p-Smad3 in PLC and Huh7 cells were detected by WB. The levels of TGF-*β*1, p-Smad2, and p-Smad3 in the I-125+TGF-*β*1 group were greatly reduced but that of E-cadherin was increased after subtracts of I-125 group. Simultaneously, the results of the IF assay (Figures 5(d), 5(e), and 5(f)) were consistent to those of the WB assay. These results suggest that TGF-*β*1 activator can reverse the I-125 seed irradiation inhibitory effect on the EMT and TGF-*β*1 signaling pathway in vitro.

### 4.5. Anticancer Role of I-125 Seed Irradiation In Vivo

**Figure 6:**
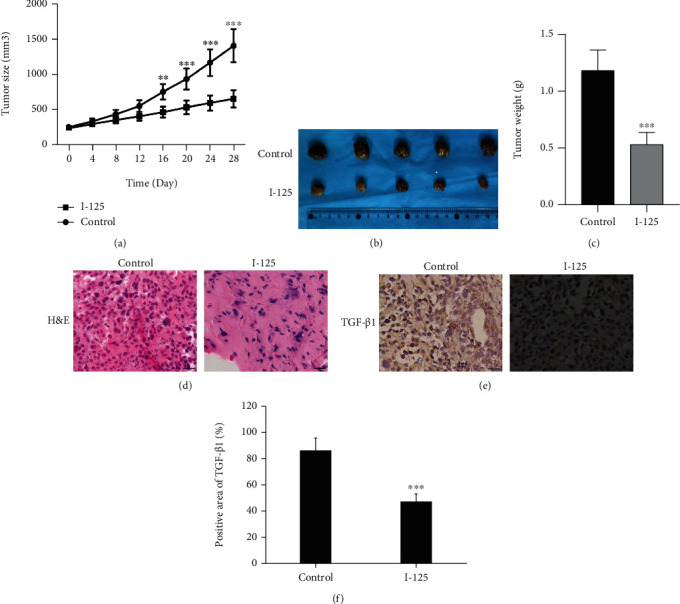
I-125 seed radiation suppressed the growth of PLC cells xenografts in mice. (a) Growth curves presented the volumes of xenograft tumor treated with I-125 seed radiation. (b) Images of the harvested tumors. (c) Weight of harvested tumors was measured 28 days after treatment. H & E staining (d) and immunohistochemistry (e). The positive expression of TGF-*β*1 in control and I-125 was quantitatively analyzed (f). The expression of data as mean ± SD (*n* = 5). ^∗∗^*P* < 0.01 vs. the control group.

The size of tumors in I-125 implanted nude mice was increased compared with the control group (Figures 6(a)–6(c)), that is, I-125 seed irradiation greatly restrained the rise of tumor volume and tumor weight, and the difference was statistically significant from 16 days after I-125 seed implantation. Consistently, H & E staining showed that the cell density of the I-125 group was greatly weakened (less emerged), and the nuclear fragmentation and necrosis were significantly increased by comparing with the control group (Figure 6(d)). In addition, I-125 seed irradiation attenuates the expression of TGF-*β*1 in xenografts (Figures 6(e) and 6(f)), which also indicate the I-125 influence the TGF-*β*1-triggered EMT signaling. Even though TGF-*β*1 is very decisive in initiation of cell proliferation, migration, and EMT, but it needs to further study because dose, timing, and many other factors in vivo may influence the pathway much more than the study in vitro.

## 5. Discussion

Radiotherapy is one of the three commonly used methods for cancer treatment, and about 60% of cancer patients receive radiotherapy. However, due to severe side effects of traditional radiotherapy, Barcelona guidelines do not recommend radiotherapy for HCC. Radioactive seed implantation therapy is to implant I-125 seed into tumor under the guidance of imaging in order to kill tumor cells and to achieve the purpose of tumor treatment. Compared with traditional radiotherapy, it has the characteristics of high target rates and sharp peripheral dose drop, which can significantly decrease the incidence of adverse effects. In addition, several studies have shown that continuous low-dose radiotherapy with I-125 seed has stronger biological effects than conventional high-dose radiotherapy [16–18]. The merits of this therapy makes it a comprehensive treatment options for patient with advanced HCC. Nevertheless, the mechanisms of radioactive particles in treating HCC keep largely elusive. In this study, we found that the I-125 seed irradiation inhibited the proliferation, invasion, and migration of HCC cell lines. Moreover, the I-125 seed irradiation inhibited EMT of HCC cell lines by partially suppressing TGF-*β* signal pathway. Also, the I-125 seed irradiation inhibited the growth of HCC in vivo.

Although I-125 radioactive is widely believed to have a gamma ray that triggers mitophagy and a number molecular chain reactions by elevating mitochondria reactive oxygen species (ROS) [19], a group of key signaling molecules plays important roles in the progression of inflammatory disorders as well as anti-inflammation in a dose-dependent manner; thus, antitumor effects of I-125 seed with certain strength of radioactivity can be achieved by inhibiting cell proliferation, inducing apoptosis and autophagy, and arresting the cell cycle [20–22]. To better understand the I-125 seed effects on the invasion ability of PLC and Huh7 cells, the viability of PLC and Hu7 cells were detected by using a CCK8 assay; IC50 of 24 h after I-125 irradiation was counted out. The results are consistent with previous studies [23, 24]. However, we found that I-125 seed irradiation significantly restrained the proliferation of cancer cells, and this restraint effect was dose-dependent. Moreover, IC50 of PLC and Huh7 cells was 6.20 Gy and 5.39 Gy at 24 hours after irradiation. Furthermore, our results are slightly different from the IC50 reported previously. The possible reason is that different types of tumor cells have different radiosensitivity.

According to the previous research, high-dose radiation drives the migration and invasion of tumor cells. In contrast, continuous low-dose radiation of I-125 seed inhibits the migration and invasion of tumor cells [17]. The outcomes indicated I-125 seed irradiation significantly inhibited the migration and invasion of HCC cells below IC50. According to the outcomes, the inhibitory effect of I-125 seed on the migration and invasion of PLC and Huh7 cells is brought not only by radioactive cytotoxicity but also by DNA damage or modification on the site of specific regulation of genes transcription [25]. Our results were largely consistent with previous studies, and the change of the gene expression by I-125 seed irradiation inhibited the invasion of nasopharyngeal carcinoma and gastric cancer [26, 27].

EMT is considered to be important for tumor cells acquiring the ability of migration and invasion [5]. Through the EMT process, the expression of epithelioid properties related proteins (such as E-cadherin) decreased and the expression of interstitial properties-related proteins (such as N-cadherin and vimentin) increased [6]. According to the past researches, I-125 seed irradiation downregulates the expression of N-cadherin while upregulating the expression of E-cadherin in lung cancer cells and glioma cells, indicating that the I-125 seed irradiation inhibits the occurrence of EMT in those cells [10, 28]. In our study, the morphology of EMT has been observed, and shown that I-125 also promotes the expression of E-cadherin and reduces the expression of N-cadherin and vimentin, suggesting that I-125 seed irradiation inhibits the EMT of PLC and Huh7 cells.

TGF-*β*1 belongs to the TGF-*β* family, functioning vital in tumor cell proliferation, differentiation, invasion, and apoptosis [29]. TGF-*β*1 binds to TGF-*β* II receptor on cell membrane to convert Smad2 and Smad3 into active p-Smad2 and p-Smad3. Subsequently, p-Smad2 and p-Smad3 enter the nucleus to regulate the transcription and expression of corresponding target genes, which induce the EMT and enhance the migration and invasion of tumor cells in the final [7, 30]. According to the past researches, radiotherapy inhibits the expression of TGF in tissues, and the inhibitory effect of low-dose radiotherapy is stronger than that of conventional high-dose radiotherapy [31, 32]. The outcomes show that I-125 seed irradiation also restrained the expression of TGF-*β*1. In addition, we found that I-125 seed irradiation inhibited the expression of p-Smad2 and p-Smad3, while the expression of Smad2 and Smad3 was not impacted by I-125 seed irradiation, indicating that I-125 seed irradiation restrained the activity of the TGF-*β*1/Smad pathway.

However, whether the TGF-*β*1/Smad signaling pathway function is vital in inhibiting the invasion and metastasis of PLC and Huh7 cells by I-125 seed remains to be further studied. According to the past studies, TGF-*β*1 activator can activate the TGF-*β*1 signaling pathway, promote epithelial-mesenchymal transition, and then promote tumor invasion and metastasis [8, 33]. Therefore, we studied the role of the TGF-*β*1 activator in the inhibitory of the I-125 seed-induced cell migration. After adding 10 ng/ml TGF-*β*1 activator, we found that the number of cell migration and invasion in the I-125+TGF-*β*1 group was highly larger than that in the I-125 group. At the same time, we found that by comparing with the I-125 group, the expression of TGF-*β*1 and p-Smad3 in the I-125+TGF-*β*1 group was greatly enhanced, while the expression of the E-cadherin was decreased. These results indicate that the TGF-*β*1 activator can at least partially block the I-125 seed irradiation-induced cell migration inhibition by restoring p-Smad3 protein level and further prove that the I-125 seed irradiation inhibits cell migration of HCC cells by suppressing the TGF-*β*1/Smad signal. We revealed a new mechanism of the I-125 seed irradiation inhibiting the development of HCC.

## 6. Conclusion

**Figure 7:**
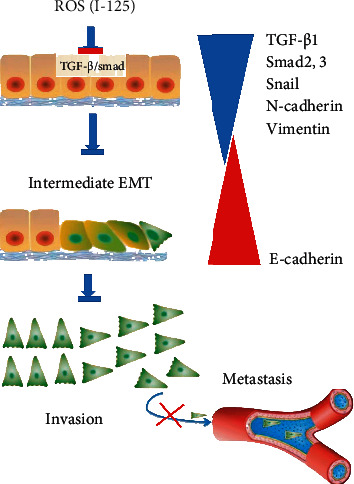
Graphical abstract: TGF-*β* signaling mediates EMT. (a) TGF-*β* is an important player in the activation of EMT, which is characterized by downregulation of epithelial markers and upregulation of mesenchymal markers. TGF-*β* via either Smad or non-Smad signaling can promote EMT. The polyhedral fibroblasts were replaced by typical cobblestone like epithelioid cells when EMT takes place. The certain radiation of gamma-ray dose from I-125 seed increased production of reactive oxygen species (ROS) in almost tested cells, which shows downregulation of TGF-*β*1, Smad2, Snail, N-cadherin, and vimentin in our tested cells by RT-PCR and WB assay. Inhibition of the TGF-*β*1/Smad signaling pathway causes the inhibition of invasion and metastasis. The diagram has been partly adapted from the concepts of ref. 34, 35.

This study shows that radioactive I-125 seed can restrain the proliferation, migration, and invasion of HCC cells. The inhibitory effect of I-125 seed on EMT was attributed to the suppressing of the TGF-*β*1 signaling pathway (Figure 7: graphical abstract).

## Figures and Tables

**Table 1:** Sequences of PCR primers for target gene detection.

Gene	Sequence (5′->3′)	Length (bp)	Tm^1^ (°C)	Product size (bp)
E-cadherin-F	ATTTTTCCCTCGACACCCGAT	21	61.5	109
E-cadherin-R	TCCCAGGCGTAGACCAAGA	19	61.9	
N-cadherin-F	TGCGGTACAGTGTAACTGGG	20	61.5	123
N-cadherin-R	GAAACCGGGCTATCTGCTCG	20	62.7	
Vimentin-F	GACGCCATCAACACCGAGTT	21	62.4	98
Vimentin-R	CTTTGTCGTTGGTTAGCTGGT	21	62.5	
TGF-*β*1-F	CAATTCCTGGCGATACCTCAG	21	60.2	86
TGF-*β*1-R	GCACAACTCCGGTGACATCAA	21	62.9	
Smad2-F	CCGACACACCGAGATCCTAAC	21	61.9	125
Smad2-R	GAGGTGGCGTTTCTGGAATATAA	23	60.1	
Snail-F	TCGGAAGCCTAACTACAGCGA	21	62.7	140
Snail-R	AGATGAGCATTGGCAGCGAG	20	62.6	

Internal standard: GAPDH, F: TCAAGAAGGTGGTGAAGCAGG, R: AGCGTCAAAGGTGGAGGAGTG. [1]Tm: temperature.

## Data Availability

All data generated or analyzed during this study are included in this article.
